# Effects of Proton Pump Inhibitors on the Gastrointestinal Microbiota in Gastroesophageal Reflux Disease

**DOI:** 10.1016/j.gpb.2018.12.004

**Published:** 2019-04-25

**Authors:** Yi-Chao Shi, Shun-Tian Cai, Ya-Ping Tian, Hui-Jun Zhao, Yan-Bing Zhang, Jing Chen, Rong-Rong Ren, Xi Luo, Li-Hua Peng, Gang Sun, Yun-Sheng Yang

**Affiliations:** 1 Department of Gastroenterology and Hepatology, Institute of Digestive Diseases, Chinese PLA General Hospital, Beijing 100853, China; 2 School of Medicine, Nankai University, Tianjin 30071, China; 3 Core Laboratory of the Translational Medical Center, Chinese PLA General Hospital, Beijing 100853, China; 4 Realbio Genomics Institute, Shanghai 200050, China

**Keywords:** Microbiota, Gastroesophageal reflux disease, Proton pump inhibitors, Gastric mucosal, Fecal

## Abstract

**Proton pump inhibitors** (PPIs) are commonly used to lessen symptoms in patients with **gastroesophageal reflux disease** (GERD). However, the effects of PPI therapy on the gastrointestinal **microbiota** in GERD patients remain unclear. We examined the association between the PPI usage and the microbiota present in **gastric mucosal** and **fecal** samples from GERD patients and healthy controls (HCs) using 16S rRNA gene sequencing. GERD patients taking PPIs were further divided into short-term and long-term PPI user groups. We showed that PPI administration lowered the relative bacterial diversity of the gastric microbiota in GERD patients. Compared to the non-PPI-user and HC groups, higher abundances of Planococcaceae, Oxalobacteraceae, and Sphingomonadaceae were found in the gastric microbiota from the PPI-user group. In addition, the *Methylophilus* genus was more highly abundant in the long-term PPI user group than in the short-term PPI-user group. Despite the absence of differences in alpha diversity, there were significant differences in the fecal bacterial composition of between GERD patients taking PPIs and those not taking PPIs. There was a higher abundance of Streptococcaceae, Veillonellaceae, Acidaminococcaceae, Micrococcaceae, and Flavobacteriaceae present in the fecal microbiota from the PPI-user group than those from the non-PPI-user and HC groups. Additionally, a significantly higher abundance of *Ruminococcus* was found in GERD patients on long-term PPI medication than that on short-term PPI medication. Our study indicates that PPI administration in patients with GERD has a significant effect on the abundance and structure of the gastric mucosal microbiota but only on the composition of the fecal microbiota.

## Introduction

Gastroesophageal reflux disease (GERD) is defined as the abnormal reflux of the contents of the stomach and duodenum that causes troublesome symptoms such as heartburn, regurgitation, or complications (*e.g.*, noncardiac chest pain, laryngitis, asthma, and cough). The prevalence of GERD is 10%–20% in the Western world and 5.2%–18.3% in Asia [1].

Proton pump inhibitors (PPIs) are the most effective first-line treatment for GERD patients. PPIs do not effectively eradicate this disease, and some patients may require this treatment for life as continuous maintenance therapy or only when symptoms are present. Maintenance therapy is not a minor issue because symptom recurrence has been described when PPI therapy is discontinued, and these symptoms may severely impair quality of life.

In general, PPI administration is considered safe, and there have been few reports of serious adverse events. However, long-term PPI use may result in several side effects, such as decreased bone metabolism, increased incidence of bone fractures, iron deficiency anemia, vitamin B12 deficiency, hypomagnesemia, and pneumonia [2], [3], [4]. Recent studies have also demonstrated that long-term use of PPIs increases the risk of infections by enteric bacteria such as *Clostridium difficile*, *Campylobacter* spp., *Shigella* spp., and *Salmonella* spp. [5], [6], [7], [8], [9], [10].

PPIs have been reported to substantially increase the abundance of commensals in the upper gastrointestinal (GI) tract, decrease microbial diversity and lower the abundance of commensals in the gut. At the family level, *Streptococcaceae* is significantly increased in PPI-users [11]. Imhann et al. [12] examined 16S rRNA gene sequences to detect profound changes in the gut microbiota of PPI-users from 1815 individuals. In PPI-users, the relative abundances of 20% of bacterial taxa, such as the genera *Staphylococcus*, *Streptococcus*, and *Enterococcus* as well as *Escherichia coli* species, were significantly increased compared with the abundances in samples from non-users. A study by Tsuda et al. [13] revealed that there was no significant difference in bacterial diversity in the gastric fluid microbiota between PPI-users and PPI-non-users. However, the beta diversity of the gastric fluid microbiota significantly increased after PPI treatment [13]. Another study by Amir et al. [14] also demonstrated that the beta diversity of the gastric fluid microbiota in subjects increased after 8 weeks of PPI therapy. Furthermore, *H. pylori* was found to be a minor bacterium in gastric luminal samples in a study by Tsuda et al. [13], whereas a separate study identified this organism as a dominant bacterium in gastric mucosal samples from *H. pylori-*infected patients, as expected [15].

It is believed that PPI-mediated disruption of the normal gastric microenvironment and direct targeting of bacterial and fungal proton pumps are the potential mechanisms by which PPIs affect gastric bacterial composition. While the relationship between PPI use, especially continuous PPI use, and the gastric/gut microbiota is not fully understood, it is also unclear whether long-term PPI use affects the gastric and gut microbiota.

In our study, we investigated the effects of PPIs on the microbiota of gastric mucosal and fecal specimens of GERD patients. We used 16S rRNA gene sequencing to evaluate the characteristics of the GERD microbiota and further clarify the association between PPI use and the gastric/fecal microbiota community.

## Results

### 16S rRNA gene sequencing of gastric mucosal and fecal samples

To characterize the effects of PPIs on the gastric mucosal and fecal microbiota, we performed 16S rRNA gene sequencing analysis and compared the microbial community structures between healthy controls (HCs) and GERD patients. The clinical characteristics of the three groups, namely, GERD patients with and without PPI use and HCs, are shown in Table 1. The mean age, sex, and BMI were not significantly different between HCs and GERD patients with or without PPI use (*P* > 0.05; Kruskal–Wallis test).

**Table 1:** **Clinical characteristics of the subjects who participated in the study**

**Sample**	**Parameter**	**HC**	**Non-PPI-user**	**PPI user**	***P* value**
Gastric	No. of subjects	5	10	20	
	PPI therapy duration (months)	–	–	13 (3–120)	–
	Age (mean ± SD)	47.4 ± 7	54.2 ± 11	49.05 ± 13.3	0.469
	Sex (male/female)	3/2	6/4	9/11	0.679
	BMI (kg/m^2^)	24.6 ± 4	27.3 ± 7	24.9 ± 6.2	0.574

Fecal	No. of subjects	15	15	25	
	PPI therapy duration (months)	–	–	16 (2–120)	
	Age (mean ± SD)	45.5 ± 10	52.6 ± 13	46.9 ± 10.8	0.184
	Sex (male/female)	7/8	9/6	12/13	0.628
	BMI (kg/m^2^)	23.8 ± 6	26.6 ± 8	25.0 ± 5.3	0.480

We obtained raw sequencing reads, and after filtering and removing the low-quality sequences, clean reads were retained for further analyses with an average of 44,254 and 45,632 sequences per sample for the mucosal and fecal microbiota, respectively. Rarefaction and rank abundance analyses suggested that sufficient sequencing information was achieved for all samples. Sequence clustering analysis yielded a total of 337 and 244 core operational taxonomic units (OTUs) (phylotypes) in the gastric mucosal and fecal microbiota, respectively.

### Characteristics of microbial diversity in GERD patients with PPI use

**Figure 1:**
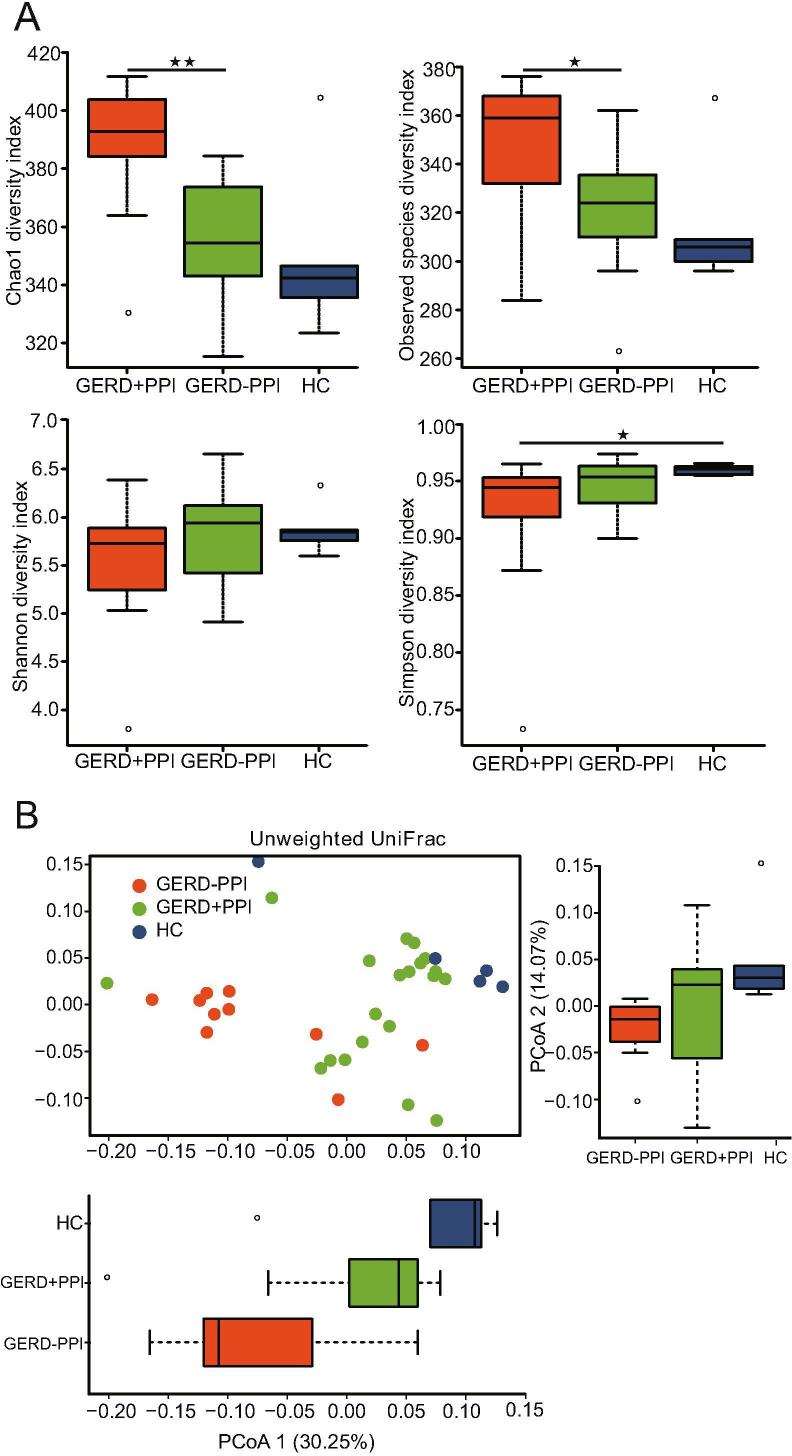
**Characteristics of gastric mucosal microbial diversity in GERD patients with PPI use** **A.** Four metrics of alpha diversity (Chao1 estimator richness, observed species, Shannon index, and Simpson index) were calculated in gastric mucosal samples. Two-tailed Wilcoxon rank sum tests were performed to assess differences between the non-PPI-user, PPI-user, and HC groups. The middle line in the box plot represents the median value, and the box is drawn from the 25% to 75% quartiles. Whiskers show the minimum and maximum values, and the ends of the whiskers represent the nonoutlier range. **B.** PCoA of an unweighted UniFrac analysis plot based on the relative taxa abundance in the gastric mucosal microbiota of GERD patients and HCs. Each symbol represents a sample. PPI, proton pump inhibitor; GERD, gastroesophageal reflux disease; HC, healthy control; PCoA, principal component analysis.

To investigate how PPIs affect the composition of the gastric mucosal, alpha diversity indexes, including species richness and evenness, were calculated for the gastric mucosal biopsy samples (Figure 1A). The HC group showed no significant difference in bacterial richness (Chao1, *P* = 0.076; observed species, *P* = 0.098; Wilcoxon test) and significantly higher community diversity (Simpson, *P* = 0.024) than that of non-PPI-users (Figure 1A). However, the non-PPI-user group had significantly higher Chao1 (*P* = 0.001; Wilcoxon test) and observed species (*P* = 0.013; Wilcoxon test) indexes than those of the PPI-user group (Figure 1A). In addition, no significant differences in the Shannon index (*P* > 0.05 for all comparisons; Wilcoxon test) were found among the groups. Overall, there was no significant difference in the alpha diversity of the gastric mucosal microbiota between HCs and the PPI-user group (Figure 1A).

Principal component analysis (PCoA) was performed to identify discrepancies in the gastric mucosal microbiome associated with PPI medication (Figure 1B). As expected, samples from GERD patients formed a cluster that was distinct from the cluster of HC samples, and PCoA1 and PCoA2 accounted for 30.25% and 14.07% of the variance, respectively (Figure 1B). These differences were further investigated by an analysis of similarities test (ANOSIM, *P* = 0.001) (Figure S1).

**Figure 2:**
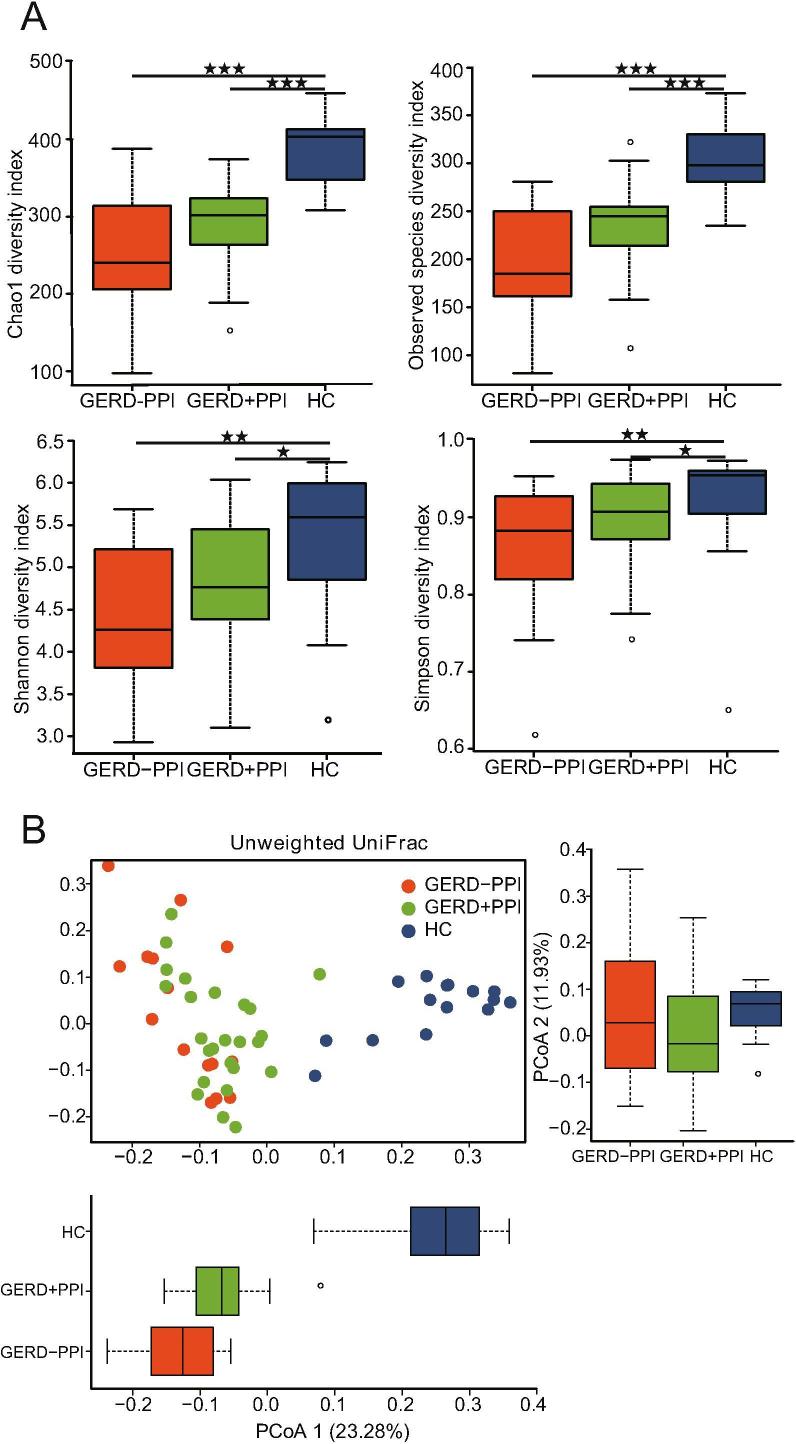
**Characteristics of fecal microbial diversity in GERD patients with PPI use** **A.** Alpha diversity plots of Chao1 estimator richness, observed species, Shannon index, and Simpson index measures for fecal samples in the non-PPI-user, PPI-user, and HC groups. Wilcoxon rank sum tests were used to determine the significance of the differences between groups. **B.** PCoA of unweighted UniFrac analyses of the differences in the fecal microbiota between non-PPI-user or PPI-user GERD patients and the HC group. PC1 and PC2 represent the two highest discriminating axes. The corresponding subject’s number is adjacent to each symbol.

The alpha diversity of the fecal samples was assessed with the Shannon index, Chao1 index, and number of observed species. The HC group had significantly greater bacterial richness (Chao1 and observed species) than that of GERD patients with or without PPI use and had higher community diversity (Shannon and Simpson indexes) than that of the non-PPI-user and PPI-user groups (Figure 2A). The alpha diversity of the gut microbiota had no significant difference between GERD patients with and without PPI use (Figure 2A). An analysis of beta diversity calculated using unweighted UniFrac distances revealed that the gut bacterial microbiota of GERD patients with or without PPI use were clustered apart from that of HCs in PCoA1 (23.28%) and PCoA2 (11.93%) (Figure 2B). However, overrepresentation of the gut microbiome of fecal bacteria in the non-PPI-user group and PPI-user group was not significantly different, as determined by tax_all difference analysis (Figure S2).

### Characteristics of the gastric mucosal microbial composition in GERD patients with PPI administration

**Figure 3:**
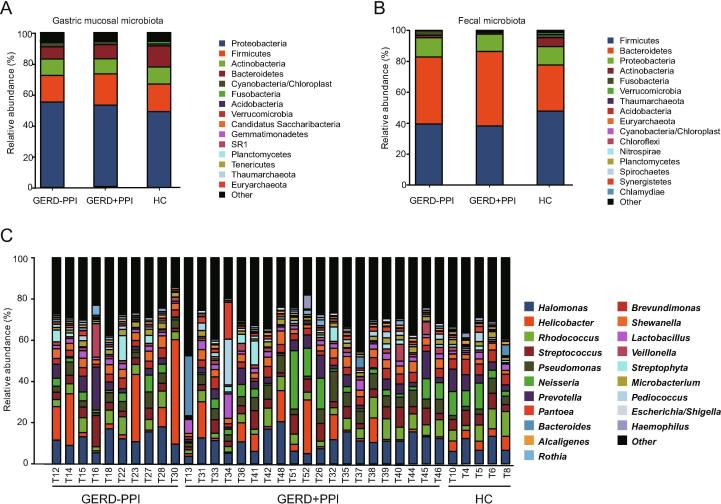
**Characteristics of the microbial composition in GERD patients with PPI use** **A.** Relative abundance of the dominant bacteria at phylum level in the gastric mucosal microbiota of GERD patients with or without PPI use and the HC group. **B.** Relative abundance of the dominant bacteria at phylum level in the fecal microbiota of GERD patients with or without PPI use and the HC group. **C.** Relative abundance of the top 35 dominant bacteria at genus level in the gastric mucosal microbiota of GERD patients with or without PPI use and the HC group.

Taxonomic compositions of the microbiota obtained from the gastric mucosal samples of all the subjects were analyzed at the phylum and genus levels. The five dominant, most abundant phyla in the gastric mucosal microbiota were Proteobacteria, Firmicutes, Bacteroidetes, Actinobacteria, and Fusobacteria, accounting for 92.7%−94.7% of all reads (Figure 3A). The five dominant, most abundant phyla in the fecal microbiota were Firmicutes, Bacteroidetes, Proteobacteria, Actinobacteria, and Fusobacteria, accounting for 97.5%−99.7% of all sequence reads (Figure 3B). In addition, the gastric microbiota composition of the major genera in the HCs, non-PPI-user, and PPI-user groups among the different groups are shown in Figure 3C. Among the 143 genera identified in total, *Halomonas* (10.7%), *Helicobacter* (7.7%), *Rhodococcus* (5.9%), *Neisseria* (5.4%), *Streptococcus* (5.2%), *Pseudomonas* (5.0%), *Prevotella* (4.9%), *Brevundimonas* (4.1%), *Shewanella* (3.5%), *Lactobacillus* (2.6%), *Veillonella* (2.0%), and *Microbacterium* (2.0%) were the 12 most abundant genera (Figure 3C).

### Variations of the microbiota in GERD patients with PPI use

**Figure 4:**
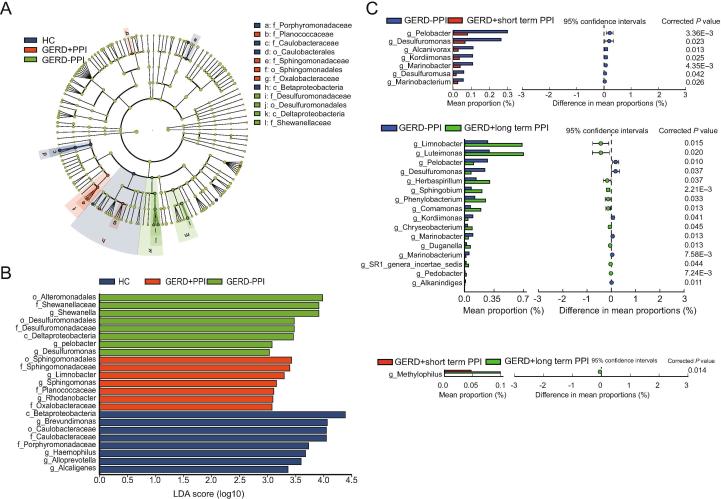
**Variations in the gastric mucosal microbiota in GERD patients with PPI use** **A.** Cladogram derived from LEfSe analysis of metagenomic sequences of gastric mucosal samples from HCs and GERD patients. The prefixes “p”, “c”, “o”, “f”, and “g” indicate the phylum, class, order, family, and genus, respectively. **B.** LEfSe comparison of the microbiota in gastric samples from GERD patients with or without PPI use and the HC group. Enriched taxa in samples from GERD patients and HCs with different classification levels with an LDA score >3.0 are shown. **C.** Extended error bar plots showing functional properties that differ between the gastric mucosal microbiota of non-PPI-users, short-term PPI-users, and long-term PPI-users. LEfSe, linear discriminant effect size; LDA, linear discriminant analysis.

Linear discriminant effect size (LEfSe) analysis and cladograms were used to analyze the gastric mucosal bacterial community structure. Linear discriminant analysis (LDA) was used to estimate the difference in the effect size of each taxon among the HC, non-PPI-user, and PPI-user groups. The bacterial taxa with significantly higher abundances in the HC group were Caulobacteraceae and Porphyromonadaceae. In contrast, Desulfuromonadaceae, and Shewanellaceae were higher in the non-PPI-user group, whereas Planococcaceae, Oxalobacteraceae, and Sphingomonadaceae were higher in the PPI-user group (Figure 4A, B).

We further analyzed the differences in gastric mucosal bacterial community structure between GERD patients with or without PPI administration. According to the duration of PPI administration, we divided the PPI-user group into patients who received short-term PPI treatment (average time = 6 [3–12] months) and those who received long-term PPI treatment (average time = 18 [12–120] months). There were no differences among the non-PPI-user, short-term PPI-user, and long-term PPI-user groups with respect to sex, age, and BMI (data not shown). Extended error bar plots were constructed to determine the mean gastric mucosal microbiota differences among the non-PPI-user, short-term PPI-user, and long-term PPI-user groups. At the genus level, samples from the short-term PPI-user group had significantly lower levels of *Pelobacter*, *Desulfuromonas*, *Alcanlvorax*, *Kordiimonas*, *Desulfuromusa*, *Marinobacterium*, and *Marinobacter* compared with the samples from GERD patients without PPI administration (*P* < 0.05; Welch’s t-test). The relative abundances of *Luteimonas*, *Limonobacter*, *Herbaspirillum*, *Sphingobium*, *Phenylobacterium*, *Comamonas*, *Chryseobacterium*, *Duganella*, *Pedobacter*, and *SR1 genera incertae sedis* were higher in the long-term PPI-user group than those in the non-PPI-user group (Figure 4C). The relative abundances of the genera *Pelobacter*, *Desulfuromonas*, *Alkanindiges, Koridiimonas, Marinobacterium*, and *Marinobacter* were significantly lower in the long-term PPI-user group than those in the non-PPI-user group. There was a higher abundance of the *Methylophilus* genus in GERD patients with long-term PPI use compared with the abundance in the short-term PPI-user group (Figure 4C).

**Figure 5:**
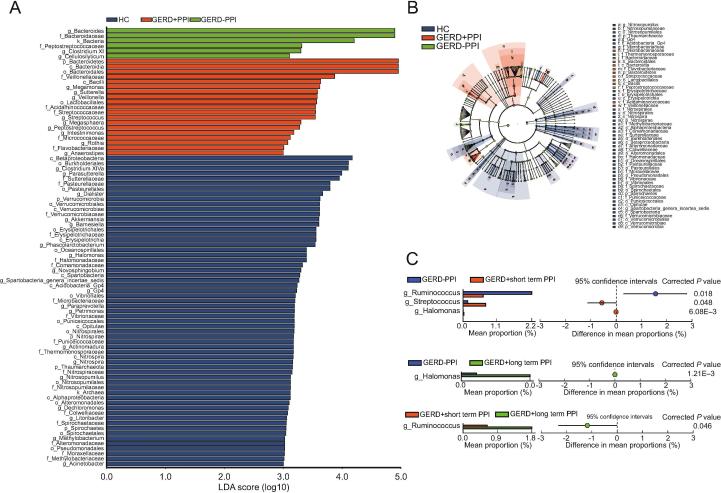
**Variations in the fecal microbiota in GERD patients with PPI use** **A.** LEfSe comparison of the microbiota in fecal samples from GERD patients with or without PPI use and the HC group. Enriched taxa in samples from GERD patients and HCs with different classification levels with an LDA score >3.0 are shown. **B.** Cladogram plotted based on LEfSe analysis showing the taxonomic levels represented by rings, with phyla in the outermost ring and genera in the innermost ring. Each circle represents a member within that level. The taxa at each level are colored according to abundance (*P* < 0.05; LDA score >3). **C.** Extended error bar plots showing significantly different microbiota between non-PPI-user, short-term PPI-user, and long-term PPI-user with an effect size ≥1%.

The overrepresentation of fecal bacteria of GERD patients with or without PPI use, and HCs is depicted in a cladogram. LEfSe analysis (LDA ≥3) revealed a significantly higher relative abundance of Sutterellaceae, Pasteurellaceae, Methylobacteriaceae, Halomonadaceae, Comamonadaceae, Thermomonosporaceae, Nitrosopumilaceae, Vibrionaceae, Erysipelotrichaceae, Microbacteriaceae, Puniceicoccaceae, Nitrospiraceae, Colwelliaceae, Spirochaetaceae, Alteromonadaceae, Moraxellaceae, and Verrucomicrobiaceae in HC samples compared with GERD patient samples (Figure 5A, B). In contrast, the gut microbiota of the non-PPI-user group was enriched in microbes from the Bacteroidaceae and Peptostreptococcaceae families (Figure 5A, B). In addition, there was a significantly higher abundance of Streptococcaceae, Veillonellaceae, Acidaminococcaceae, Micrococcaceae, and Flavobacteriaceae in the PPI-user group than that in the other groups (Figure 5A, B).

We further divided the PPI-user group into subgroups, namely, short-term PPI-user (average time = 6 [2–12] months) and long-term PPI-user (average time = 24 [12–120] months) groups, to explore differences in the fecal microbiota in GERD patients. There were no differences in the microbiota in fecal samples among the non-PPI-user, short-term PPI-user, and long-term PPI-user groups with respect to sex, age, and BMI (data not shown). Our results showed that fecal microbes such as *Halomonas* were significantly lower in the non-PPI-user group than those in the short- or long-term PPI-user groups using extended error bar plots analysis (Figure 5C). The abundance of *Ruminococcus* was significantly lower in the short-term PPI-user group than that in the non-PPI-user and long-term PPI-user groups. We determined that *Streptococcus* levels were higher in the short-term PPI-user group than those in the non-PPI-user group using a differential test and clustering analysis (Figure 5C).

### Functions of metabolism in the gastric mucosal and fecal microbiome in GERD patients with PPI use

**Figure 6:**
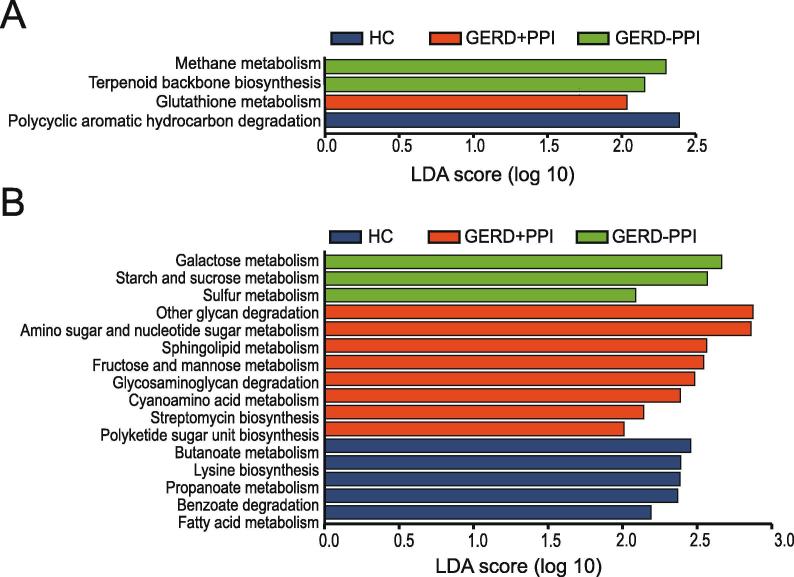
**PICRUSt analysis predicts functional composition in GERD patients with PPI use** **A.** Predicted functional composition of metagenomes based on 16S rRNA gene sequencing data from the gastric mucosal microbiota. Pathway enrichment for KEGG metabolic pathways followed by statistical comparative analysis using LEfSe were performed to determine differential enrichment between the non-PPI-user, PPI-user, and HC groups. **B.** Predicted metabolic functions of the fecal bacterial communities were generated with LEfSe based on the PICRUSt dataset, showing significantly differing abundance in GERD patients with or without PPI use and HCs.

To detect changes in microbiome metabolites induced by changes in microbiota abundance, the 16S rRNA data were annotated with metabolic pathways from the KEGG database using PICRUSt prediction analysis. KEGG metabolic pathways correlating with gastric mucosal and fecal microbiome differences among the non-PPI-user, PPI-user, and HC groups were revealed. According to the LEfSe analysis of metabolic function pathways, the gastric mucosal microbiota of the non-PPI-users included more relative methane metabolism, and terpenoid backbone biosynthesis pathways (Figure 6A). Glutathione metabolism was more prevalent in the PPI-users, whereas polycyclic hydrocarbon degradation was elevated in HCs (Figure 6A).

As shown in Figure 6B, the fecal microbiota metabolism of the non-PPI-users were more active in starch andsucrosemetabolism, galactose metabolism, and sulfur metabolism. For fecal microbiota metabolism, the pathways related to other glycan degradation, amino sugar and nucleotide sugar metabolism, sphingolipid metabolism, fructose and mannose metabolism, cyanoamino acid metabolism, glycosaminoglycan degradation, streptomycin biosynthesis and polyketide sugar unit biosynthesis were abundant in the PPI-users. Fatty acid metabolism, lysine biosynthesis, benzoate degradation, butanoate metabolism, and propanoate metabolism were more active in the HC group (Figure 6B).

## Discussion

With the development of high-throughput sequencing technology, 16S rRNA sequencing has been widely used to obtain large amounts of information regarding the stomach and fecal microbiota, but the defining characteristics of the gastric and gut microbiome associated with PPI use in GERD patients are still not fully understood.

In recent years, numerous studies have consistently demonstrated that the inhibition of gastric acid secretion induces the dysbiosis of the gastric microbiota, including affecting the composition of the intestinal microbiota and promoting gastric bacterial overgrowth [16]. The long-term use or overuse of PPIs has been demonstrated to shift the gastrointestinal microbiota toward an unhealthy state. Many studies have described the effects of PPI treatment on the gut and gastric microbiota, but yielding conflicting results. In this study, 16S rRNA gene amplicons from the gastric mucosal and fecal microbiota of GERD patients treated with PPIs were sequenced to evaluate and characterize these bacterial communities in detail. We identified significant alterations in the gastric mucosa-associated microbiota after PPI use in GERD patients.

Previous research revealed that the gastric mucosa-associated microbiota is strongly dominated by Proteobacteria, Firmicutes, Bacteroidetes, Actinobacteria, and Fusobacteria at the phylum level [17], [18], [19]. Consistent with previous studies, our results confirmed the same dominant bacterial phyla but found different genera residing in the gastric mucosa. Li et al. [19] performed 16S rRNA gene sequencing of gastric mucosal biopsies to evaluate the association between the gastric microbiota and gastritis in patients and normal individuals. The authors found that the most common bacteria included *Prevotella*, *Streptococcus*, *Neisseria*, *Porphyromonas*, and *Haemophilus* at the genus level. Furthermore, *Streptococcus* and *Prevotella* were shown to be the dominant genera in both the gastric lumen and gastric mucosa-associated microbiota [13], [17], [18], [19]. In addition, our study showed that the most abundant genera in the gastric mucosal microbiota were *Halomonas*, *Neisseria*, *Rhodococcus*, *Brevundimonas*, *Prevotella*, and *Streptococcus*. The differing abundances at the genus level in our study and previous studies may be partially attributed to different geographic and ethnic differences in the study participants.

Recent studies have indicated that contamination was present during microbiota sequencing experiments, occurring between sample collection and sequencing [20], [21], [22], [23]. To prevent contamination, sterile cryopreservation tubes treated with ultraviolet radiation were used to collect mucosal and fecal samples. During nucleic acid extraction and amplification, a negative control (ultrapure water) was included, resulting in a negative PCR. Furthermore, negative control samples (including ultrapure water and PCR amplified bank control) were used for simultaneous DNA sequencing. The results indicated that there were 696 reads of negative control batch to be collected, and most of them were unclassified except for one read confirmed to the Ruminococcaceae family (data not shown).

Interestingly, our study showed that certain halophilic bacteria and soil bacteria such as *Halomonas*, *Shewanella*, *Marinobacter*, and *Mesorhizobium* are present in the stomach, which may be due to the widely consumed traditional salted or fermented foods among Chinese populations. In 2017, Anubhav et al. [24] found that *Halomonas* and *Bradyrhizobium* exist in gastric mucosal and negatively correlate with *H. pylori* infection. In addition, Avilés-Jiménez et al. [25] found nitrogen-fixing bacteria, as well as high-salt soil bacteria, in extrahepatic bile duct cancer, including Methylophilaceae and *Nesterenkonia*, which can colonize human tissues and might also be associated with *H. pylori* infection. The presence of these microflora in the stomach may constitute colonized or translocation of upstream microflora, and the real composition and roles should be further confirmed.

Numerous studies have demonstrated that *H. pylori* colonization status in the gastric mucosal significantly alters the gastric microbiota [17]. In addition, *H. pylori* have been shown to have a protective effect on GERD. In our study, we did not find a difference in the abundance of *H. pylori* at the genus level between the patients and HCs. Therefore, we could not confirm the effects of *H. pylori* on the gastric mucosal microbiota; thus, the role of *H. pylori* in the pathogenesis of GERD patients requires further research.

Most previous studies have confirmed that the composition of mucosal microbiota can be significantly affected by the use of PPI. It has been proposed that PPI use affects the gastric mucosal microbiota by directly targeting the bacteria’s proton pumps or increasing the pH to indirectly affect the microenvironment of the microbiota [26]. One study demonstrated that PPI use reduced the abundance of *H. pylori* and increased the abundance of Fusobacteria and Firmicutes in the gastric mucosal microbiota of healthy dogs [27]. Sanduleanu et al. [28] revealed a significant increase in the abundance of fecal-like and oropharyngeal-like bacteria in the gastric mucosal in PPI use. However, Paroni Sterbini et al. [29] showed that PPI administration in dyspeptic patients had no significant effect on the composition of the gastric microbiota. In our study, the diversity and composition of the gastric microbiota in the non-PPI, PPI-use and HC groups differed in a complex manner. We observed that the non-PPI group possessed greater bacterial richness and community diversity than that of the HC group, as well as greater bacterial abundance than that of GERD patients in the PPI-use group. The differences in the patient populations enrolled in our study (GERD patients) and the study by Paroni Sterbini et al. (dyspeptic patients) may contribute to the differential effects of PPI administration on the gastric microbiota.

In previous reports, the gastric mucosa-associated microbiota demonstrated a higher alpha diversity in GERD patients than that in HCs, aligning with the results of the present study. The composition of the gastric mucosal microbiota differed between GERD patients and the HC group. At the family level, Porphyromonadaceae and Caulobacteraceae were more abundant in the HC group, while Desulfuromonadaceae and Shewanellaceae were more abundant in the non-PPI-user group. In addition, Planococcaceae, Sphingomonadaceae, and Oxalobacteraceae were more abundant in the PPI-user group.

We also found differences in bacterial richness and community diversity in the gastric mucosal microbiota between GERD patients with short- or long-term PPI use and non-PPI use. Extended error bar plots were generated to demonstrate that the long-term PPI-use group exhibited lower relative abundances of *Pelobacter*, *Desulfuromonas*, *Alkanindiges, Koridiimonas, Marinobacterium* and *Marinobacter* and higher relative abundances of *Luteimonas*, *Limonobacter*, *Herbaspirillum*, *Sphingobium*, *Phenylobacterium*, *Comamonas*, *Chryseobacterium*, *Duganella*, *Pedobacter*, *and SR1 genera incertae sedis* compared with the non-PPI-user group. The short-term PPI-user group had significantly lower levels of *Pelobacter*, *Desulfuromonas*, *Alcanlvorax*, *Kordiimonas Desulfuromusa*, *Marinobacterium*, and *Marinobacter* than did the non-PPI-user group. In addition, GERD patients with long-term PPI use had a higher abundance of *Methylophilus* than that of short-term PPI-users. Amir et al. [14] found that GERD patients with normal versus abnormal esophagi had significantly different gastric fluid bacterial compositions. Additionally, the authors found that gastric fluid communities were dramatically altered. At the family level, Erysipelotrichaceae and an unclassified family from the order Clostridiales were significantly higher in relative abundance but Comamonadaceae, Moraxellaceae, and Methylobacteriaceae were significantly lower in relative abundance in GERD patients after short-term PPI treatment [14]. Our results demonstrated that GERD patients with short-term PPI use had significantly higher abundances of Desulfuromonadaceae, Intrasporangiaceae, Alteromonadaceae, Alcanivoracaceae, and Kordiimonadaceae at the family level than those in non-PPI users (data not shown). The gastric microbiota was collected from the gastric mucosal in our study, while in the Amir et al. study, the microbiota was collected from gastric fluid. Previous studies have demonstrated that lumen and mucosa-associated microbial populations are not identical [30]. Thus, the selection of different gastric samples (gastric mucosal vs. fluid) may have contributed to the differing results.

It has been reported that PPI can alter the composition of the gut microbiota in healthy twins or in patients with GI disease (including IBD, IBS, and functional diarrhea or constipation) [11], [12]. Nevertheless, several studies have shown that PPI treatment has only minor effects on the fecal microbiome in patients with GERD [31]. Recent findings showing that although the abundance of Lactobacillus and Stenotrophomonas was low and the abundance of Haemophilus at genus level was high on the fecal microbiota of infants with GERD on PPI treatment, there were no significant changes in α- or β-diversity [31]. Our results showed that there was a significant difference in fecal bacterial richness and composition diversity between GERD patients and HCs. We found that HC fecal samples contained significantly higher abundances of such taxa as Sutterellaceae, Pasteurellaceae, Methylobacteriaceae, Halomonadaceae, Comamonadaceae, and Thermomonosporaceae at the family level, while fecal samples from non-PPI users contained higher abundances of Bacteroidaceae and Peptostreptococcaceae. Additionally, the PPI-use group contained a significantly higher abundance of the families Streptococcaceae, Veillonellaceae, Acidaminococcaceae, and Clostridiaceae. Nevertheless, another study found no significant change in diversity of the gut microbiota between PPI users and controls. A crossover trial performed by Freedberg et al. [32] showed no significant effect of PPI use on gut microbiota diversity in 12 healthy volunteers after 4 and 8 weeks of treatment compared with baseline. However, changes during PPI use, such as a lower relative abundance of Clostridiales and a higher relative abundance of Streptococcaceae and Enterococcaceae, were also found in the gut microbiota [32].

The effects of PPI use on gut microbiota diversity have been investigated by several studies. The free use of gastric acid suppression is potentially associated with an increased risk of *C. difficile*, *Campylobacter*, *Shigella*, *Salmonella*, and other enteric infections [8], [33], [34]. Studies have shown that PPIs alter specific taxa in the human gut microbiota, including increases in the abundance of Enterococcaceae, Streptococcaceae, Firmicutes, and *Lactobacillus* and decreases in the abundance of *Bacteroides* and *Clostridium cluster IV*, which cause a further decrease in gut microbiota diversity. Jackson et al. [11] collected fecal samples from 1827 twins and used 16S rRNA amplification to investigate the effects of PPI use on the gut microbiota. The authors found significantly lower microbial diversity and lower abundance in the gut microbiota of PPI users. In addition, they observed that 24 genera, including *Rothia* and *Streptococcus*, were positively associated with PPI use. Imhann et al. [12] investigated the association between the gut microbiota and PPI usage in fecal samples from 1815 individuals in the Netherlands and found that PPI use was associated with an increased abundance of the genera *Staphylococcus*, *Streptococcus*, and *Enterococcus* as well as *Escherichia coli* at the species level, and there was an increase in oral bacteria, including the genus *Rothia*, in the fecal microbiota of PPI users [12]. Despite some differing results from previous studies, we found that Streptococcaceae at the family level and *Streptococcus* at the genus level were significantly more abundant in short-term PPI users than nonusers. The genus *Streptococcus* causes severe damage to histiocytes and DNA by absorbing oxygen and releasing superoxide and hydrogen peroxide. Therefore, the family Streptococcaceae and genus *Streptococcus* are closely associated with PPI use. However, the real role of these bacterial taxa in GERD patients should be further investigated. In addition, changes in the microbiota associated with *C. difficile* in the PPI-use group of GERD patients were not detected. Although several studies have shown that PPI use is an independent risk factor for *C. difficile* infection [35], [36], other studies have not observed this relationship. Therefore, the link between *C. difficile* infection and PPI use should be further studied.

Clooney et al. [37] determined that there was no significant difference in the alpha diversity of the gut microbiome between long-term PPI-users and non-PPI-users; this outcome is in accordance with our findings (data not shown). There was only a slight difference in the gut microbiota of GERD patients between non-PPI-users and the short-term or long-term PPI-user groups. At the genus level, the abundance of *Halomonas* in the gut microbiota was significantly higher in PPI-users (short-term and long-term PPI users) than that in non-PPI-user GERD patients. The relative abundance of *Ruminococcus* was significantly lower in GERD patients with short-term PPI use than non-PPI-users or long-term PPI-users. The abundance of *Streptococcus* was lower in GERD patients without PPI treatment than in those with short-term PPI administration.

The major limitation of our study was the relatively limited sample size, which may have affected our statistical analysis and subsequent conclusions regarding risk factors. Larger studies are warranted to validate our findings, and further studies are needed to investigate the long-term influence of PPIs on the gastric and gut microbiomes in GERD patients. In addition, a previous study demonstrated that the gastric fluid microbiota had higher diversity compared with that of the gastric mucosa-associated microbiota. During gastric mucosal biopsy sampling, it is unavoidable that gastric fluid may be included, which may impact the results of gastric mucosal microflora. For example, *Streptococcus *is a diverse bacterial lineage that can occupy a myriad of environments; it has been found in the nasopharynx, oral cavity and esophagus [17]. The presence of *Streptococcus* in the gastric mucosal may be upstream community translocation or mucosal colonization. Therefore, whether *Streptococcus* colonizes the gastric mucosal still needs further verification, such as culturomics detection.

## Conclusions

Here, we report significant differences in the microbial community in the gastric mucosal and gut microbiota between GERD patients and HCs. In addition, GERD patients treated with PPI had significantly altered richness and structure of the gastric mucosal microbiota compared to non-PPI administration and only significantly altered the composition of the fecal microbiota. Indeed, long-term PPI users demonstrated a higher abundance of the *Methylophilus* genus in the gastric mucosal microbiota and significantly higher abundances of the genera *Ruminococcus* in the fecal microbiota than that of short-term PPI users. Additional analyses with larger sample sizes investigating PPI use in GERD patients are needed to validate our results and to confirm the risk factors associated with PPI use in GERD patients, especially in long-term PPI users.

## Materials and methods

### Patient enrollment and sample collection

Gastric mucosal biopsy and fecal samples from HCs and GERD patients were collected by the Department of Gastroenterology of the Chinese PLA General Hospital from June 2013 to July 2014. A total of 40 GERD patients and 15 HCs were enrolled in this study. All participants provided their fecal samples, whereas 30 GERD patients and 5 HCs provided gastric mucosal samples as well. The HCs received clinical examinations with normal results. In addition, the HCs had normal endoscopy results, tested negative for *H. pylori* infections (assessed using the ^13^C breath test or the rapid urease test), and did not use PPIs or other medications for at least 30 days prior to the start of the study. Patients with GERD had endoscopic evidence of esophagitis based on the Los Angeles classification. GERD patients who were non-PPI-users (non-PPI-user) were defined as patients who did not use PPIs before enrolling in the study, while GERD patients with PPI use (PPI-user) were defined as patients who used PPIs (*i.e.*, omeprazole, 40 mg/day). GERD patients with PPI use were classified into two patient subgroups based on the length of PPI therapy. GERD patients with short-term PPI use (short-term PPI-user) had taken PPIs for more than two months and less than one year at the time of study enrollment. GERD patients with long-term PPI use (long-term PPI-user) had taken PPIs for more than one year prior to study enrollment. Tissue biopsies from the gastric antrum were obtained during endoscopies, with fecal samples collected beforehand. The biopsies and fecal samples were immediately frozen in liquid nitrogen, then transferred to the laboratory and stored at −80 °C. All individuals were diagnosed by gastroendoscopy and did not receive any antibiotic treatment for one month prior to sample collection. The following exclusion criteria were applied: age below 18 years; a history of GI or hepatobiliary surgery; and suffering from organic GI lesions, such as ulcers and cancers.

This study was undertaken with the approval of the Chinese PLA General Hospital Ethics Service Committee. All experiments were performed in accordance with the approved guidelines. All individuals enrolled in this study provided their informed consent.

### DNA extraction and PCR amplification

The QIAamp DNA Mini Kit (QIAGEN, Valencia, CA, USA) combined with the bead-beating method was used to extract total DNA. After agitation with a bead beater (Fast Prep FP120 instrument, Qbiogene, Carlsbad, USA), bacterial DNA was extracted according to the manufacturer’s instructions. Bacterial DNA samples were stored at −80 °C prior to sequencing.

To characterize the taxonomic profiles of the gastric bacterial microbiota, we designed universal primers to sequence the V4 region of the 16S rRNA gene, targeting most bacteria (F: 5′-GTGCCAGCMGCCGCGGTAA-3′, R: 5′-GGACTACHVGGGTWTCTAAT-3′). The following thermal cycling conditions were applied: initial denaturation at 98 °C for 1 minute; followed by 35 cycles of denaturation at 98 °C for 10 seconds, annealing at 50 °C for 30 seconds, and elongation at 72 °C for 60 seconds; with a final incubation at 72 °C for 5 minutes. Amplifications were performed in 25-μl reactions with 50 ng of template DNA. Normalized equimolar concentrations of PCR products were then pooled and sequenced using the Illumina MiSeq PE-300 platform (Illumina, San Diego, CA, USA). Barcodes and sequencing primers were trimmed before assembly.

### 16S rRNA gene sequencing data processing

Paired-end sequence reads were assembled using FLASH (http://ccb.jhu.edu/software/FLASH/). QIIME (1.9.1) with the default setting was used for quality filtering [38]. The trimmed sequences were then chimera-filtered, singletons were discarded and the resulting sequences were assigned to OTUs (cutoff of 3% dissimilarity in 16S rRNA gene sequences) using the UPARSE pipeline (http://drive5.com/uparse/) [39]. Representative sequences for each OTU were aligned against the nonredundant SILVA database (version 123) using the Mothur algorithm. Alpha and beta diversity analyses were analyzed using identified OTUs and UniFrac distances, respectively, as implemented in QIIME.

### Functional prediction of the gastric bacterial microbiota

PICRUSt (1.0.0) was performed using the online version of Galaxy [40] (http://huttenhower.sph.harvard.edu/galaxy/root, version 1.0.0). 16S rRNA data in the form of a BIOM format table were selected by mapping all 16S reads to references in the Greengenes tree with OTUs assigned at 97% identity. The obtained OTU table was normalized by 16S rRNA copy number, and metagenomes were predicted from the KEGG catalog. Moreover, the significant microbial functional properties associated with GERD patients and HCs were analyzed by the LEfSe (1.0) method [41]. The threshold of the linear discriminant was set at 2.0. The average NSTI score of the gastric mucosal and fecal microbiota samples was 0.055 (data not shown).

### Statistical analysis

Statistical analyses of data were performed using the R packages Stats and Vegan. Significant differences in alpha diversity were assessed using the Wilcoxon rank sum test. The differences between microbial communities were determined by PCoA using the weighted UniFrac dissimilarity distance metric. The analysis of similarities (ANOSIM) test was performed with the Vegan package in R (version 2.15.3). The STAMP program is a statistical/econometric software package for time-series models with unobserved components, such as trends (available at http://stamp-software.com/). Using the STAMP program, we generated extended error bar plots to show that some properties differed in GERD patients using certain filter parameters (ratio of effect proportion ≥2, difference between proportion ≥1, *P* = 0.05).

The differentiating features of gastric mucosal and fecal microbiota composition and function were determined using the LEfSe algorithm method (available at http://huttenhower.sph.harvard.edu/galaxy). A significant alpha at 0.05 based on the Kruskal–Wallis test and an LDA score ≥3 in LEfSe was employed to identify differing features between microbial communities [41].

## Data and materials availability

All sequencing data are publicly available in the BioProject database under research ID PRJNA477423 and the SRA accession number SRP151219 (https://www.ncbi.nlm.nih.gov/sra/SRP151219).

## Authors’ contributions

YCS analyzed the data and drafted the manuscript; STC collected the samples and analyzed the data; YPT analyzed the data; YBZ, JC, HJZ, XL, and RRR assisted with analysis; LHP and GS assisted with design and revised the manuscript for important intellectual content; and YSY designed and supervised the study. All authors read and approved the final manuscript.

## Conflict of interest

The authors have declared no competing interests.

## Supplementary material

Supplementary Figure S1 **Results from the analysis of similarities (ANOSIM) between communities (Bray-Curtis dissimilarity) in the gastric mucosal microbiota of the non-PPI-user, PPI-user, and HC groups**

Supplementary Figure S2 **Differences in the distribution of the top 20 most abundant taxa based on principal component analysis in fecal samples from the non-PPI-user, PPI-user, and HC groups**
