# Supplementary figures and images for: Effects of Proton Pump Inhibitors on the Gastrointestinal Microbiota in Gastroesophageal Reflux Disease
Source: Genomics Proteomics Bioinformatics. 2019 Apr 25;17(1):52–63. doi: 10.1016/j.gpb.2018.12.004 (PMC6520915; doi:10.1016/j.gpb.2018.12.004)

$R = 0.418$ ,  $P = 0.001$

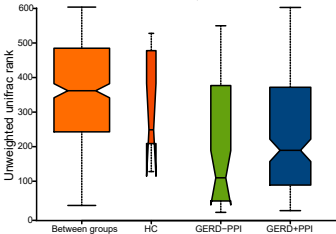

Supplement: Supplementary Figure S1 — Results from the analysis of similarities (ANOSIM) between communities (Bray-Curtis dissimilarity) in the gastric mucosal microbiota of the non-PPI-user, PPI-user, and HC groups [file mmc1.pdf]

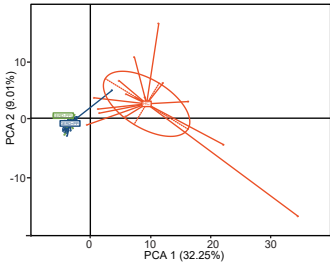

Supplement: Supplementary Figure S2 — Differences in the distribution of the top 20 most abundant taxa based on principal component analysis in fecal samples from the non-PPI-user, PPI-user, and HC groups [file mmc2.pdf]
